# The Sustained Attention Paradox: A Critical Commentary on the Theoretical Impossibility of Perfect Vigilance

**DOI:** 10.1111/cogs.70061

**Published:** 2025-04-07

**Authors:** Benjamin T. Sharpe, Ian Tyndall

**Affiliations:** ^1^ Department of Psychology and Criminology, Institute of Psychology, Business, and Human Sciences University of Chichester

**Keywords:** Attention, Cognitive limitations, Sustained attention, Human–technology systems, Vigilance decrement

## Abstract

The human capacity for sustained attention represents a critical cognitive paradox: while essential for numerous high‐stakes tasks, perfect vigilance is fundamentally impossible. This commentary explores the theoretical impossibility of maintaining uninterrupted attention, drawing from extensive interdisciplinary research in cognitive science, neuroscience, and psychology. Multiple converging lines of evidence demonstrate that sustained attention is constrained by neural, biological, and cognitive limitations. Neural mechanisms reveal that attention operates through rhythmic oscillations, with inherent fluctuations in frontoparietal networks and default mode network interactions. Neurochemical systems and cellular adaptation effects further underscore the impossibility of continuous, perfect vigilance. Empirical research across domains—including aviation, healthcare, industrial safety, and security—consistently demonstrates rapid declines in attention performance over time, regardless of individual expertise or motivation. Even elite performers like military personnel and experienced meditators exhibit inevitable attention lapses. This paper presents an argument against traditional approaches that seek to overcome these limitations through training or willpower. Instead, it advocates for designing human–technology systems that work harmoniously with cognitive constraints. This requires developing adaptive automation, understanding individual and cultural attention variations, and creating frameworks that strategically balance human capabilities with technological support.

## 1 Introduction

The human capacity for sustained attention has long fascinated cognitive scientists, psychologists, and neuroscientists alike (e.g., Fiebelkorn & Kastner, 2019; Hancock, 1989; Kahneman, 1973; Lutz, Slagter, Dunne, & Davidson, 2009; Mackworth, 1948; M. Sarter, Givens, & Bruno, 2001; Posner & Rothbart, 2008; Thomson, Besner, & Smilek, 2015; Unsworth, Robison, & Miller, 2021). Our ability to maintain focused attention over extended periods is simultaneously one of our most valuable cognitive abilities and one of our most fallible. This paradox—the critical importance of sustained attention coupled with our inherent inability to maintain it indefinitely—forms the foundation of this commentary. Through examination of major theoretical frameworks and empirical findings, this commentary argues that perfect sustained attention is not merely difficult but theoretically impossible for human beings, representing an unattainable ideal that conflicts with fundamental properties of our cognitive architecture.

## 2 Theoretical foundations and historical context

The importance of sustained attention in human functioning cannot be overstated. From air traffic controllers monitoring radar screens to nuclear power plant operators supervising complex systems, from surgeons performing lengthy procedures to students attending lectures, the ability to maintain vigilance over extended periods is crucial for both safety and performance. Specifically, Mackworth's seminal studies during World War II, investigating radar operators' ability to detect subtle signals over time, established the foundational understanding that human vigilance invariably declines over time (Mackworth, 1948). This “vigilance decrement” has since been documented across countless contexts and tasks, emerging as one of the most robust findings in attention research.

While the terms sustained attention and vigilance are often used interchangeably in the literature, a nuanced examination reveals important conceptual distinctions (Robertson & O'Connell, 2012; Warm, Parasuraman, & Matthews, 2008). Following van Schie, Lammers, Fronczek, Middelkoop, and van Dijk (2021), we define vigilance as “the capability to be aware of relevant, unpredictable changes in one's environment, irrespective of whether or not such changes occur.” This definition encompasses two critical dimensions: a quantitative aspect of alertness and a temporal dimension that acknowledges the inherent fluctuation of attentional capacity over time (Posner & Rothbart, 2007). Sustained attention, by contrast, can be understood as the ability to maintain focused cognitive resources on a specific task or stimulus over an extended period (Parasuraman & Basar, 1997). Critically, our commentary argues that “perfect vigilance”—a hypothetical state of absolute, uninterrupted environmental awareness—is fundamentally impossible due to the inherent limitations of human cognitive architecture (Mackworth, 1970). This impossibility stems not from individual deficiencies but from the adaptive design of our neural and cognitive systems.

The distinction between vigilance and sustained attention is particularly evident in clinical contexts. For instance, attention deficit hyperactivity disorder manifests as a challenge in sustained attention—a difficulty in maintaining focused task engagement—whereas conditions like narcolepsy represent a more fundamental impairment of vigilance itself (Castellanos & Proal, 2012). These differences reflect underlying neurological variations, with attention disorders primarily involving prefrontal cortex and dopaminergic systems (Faraone, 2018), while vigilance disorders involve more complex neuromodulatory networks. Methodologically, measurements of sustained attention often serve as a proxy for assessing underlying vigilance capabilities. Typical experimental paradigms involve response tasks that measure an individual's ability to detect environmental changes, evaluating performance through accuracy, response speed, or both. These measurements inherently capture both the quantitative dimension of vigilance and its temporal dynamics.

More specifically, the resource theory of attention, first proposed by Kahneman (1973) and later refined by others (e.g., Navon & Gopher, 1979; Wickens, 2008, 2024), suggests that attention operates as a limited capacity resource that becomes depleted with continuous use. This framework conceptualizes attention as a finite cognitive resource that must be allocated across competing demands. When sustained attention is required, these resources are gradually consumed, leading to deteriorating performance over time. While this theory provides an intuitive explanation for the vigilance decrement, it fails to fully account for several important observations, including the rapid onset of performance decrements and the ability to quickly recover attention with brief breaks or changes in task demands.

An alternative theoretical framework, the mindlessness theory proposed by Robertson, Manly, Andrade, Baddeley, and Yiend (1997), suggests that vigilance decrements result from the automatization of responding during repetitive tasks. According to this view, the monotonous nature of sustained attention tasks leads to a shift from controlled to automatic processing, making individuals more susceptible to lapses in attention. This theory aligns with subjective experiences of “zoning out” during repetitive tasks but struggles to explain vigilance decrements in complex, engaging tasks that resist automatization. Yet more recently, the resource‐control theory proposed by Thomson et al. (2015) attempts to bridge these perspectives by suggesting that vigilance decrements reflect a reduction in executive control rather than a depletion of attention resources per se. This theory posits that maintaining focused attention requires continuous executive control to suppress competing thoughts and responses and that this control mechanism becomes fatigued over time. While this framework addresses some limitations of pure resource theories, it still fails to fully explain the inevitability of attention lapses.

## 3 Neural perspectives

Neural mechanisms provide compelling evidence for the theoretical impossibility of perfect vigilance (e.g., Reteig, van den Brink, Prinssen, Cohen, & Slagter, 2019). The locus coeruleus–norepinephrine system, crucial for attention regulation, operates in an unsustainable phasic mode during focused attention (Aston‐Jones & Cohen, 2005). The frontoparietal network, including the dorsolateral prefrontal cortex and posterior parietal cortex, exhibits systematic activation fluctuations during sustained attention tasks, correlating with performance variations (Rosenberg et al., 2016; see also Jangraw et al., 2018). Further, neural oscillations further demonstrate this impossibility. Attention operates through rhythmic pulses, with enhanced and diminished processing occurring several times per second (VanRullen, 2016). Alpha (8–12 Hz) and theta (4–8 Hz) band oscillations modulate perceptual sensitivity and cognitive processing (Fiebelkorn & Kastner, 2019), indicating that even at the millisecond scale, truly continuous attention is impossible.

The default mode network (DMN) presents another fundamental challenge. Active during rest and mind‐wandering, the DMN supports essential functions like memory consolidation and creative problem‐solving (Raichle, 2015). Its complex interplay with task‐positive networks (Dixon et al., 2017) suggests that attention fluctuations are necessary for optimal cognitive functioning. Likewise, neurochemical systems provide additional evidence. Both the cholinergic system (M. Sarter et al., 2016) and dopaminergic systems (Cools & D'Esposito, 2011) exhibit natural activity fluctuations that correlate with attentional performance. High‐resolution neuroimaging has revealed microswitches between neural states during apparent sustained attention (Vidaurre et al., 2018), while GABAergic interneurons show adaptation effects requiring periodic recovery (Ferguson & Gao, 2018). This neural fatigue at the cellular level sets fundamental limits on the duration over which precise attentional control can be maintained.

Critically, studies of individual differences in attention networks have shown that while there is considerable variation in attentional capabilities between individuals, the fundamental constraints imposed by neural architecture remain universal. Even individuals with exceptionally high attention capacity show evidence of neural fluctuations and periodic lapses in attention, suggesting that these limitations are intrinsic to the organization of the human brain rather than individual differences in cognitive capability (Rosenberg et al., 2020).

## 4 Biological and cognitive constraints

Sleep research provides additional support for the impossibility of perfect vigilance. Studies of sleep deprivation and circadian rhythms demonstrate that our capacity for sustained attention is inherently tied to biological cycles beyond our conscious control. Research has shown that even during normal wakefulness, microsleeps and attention lapses increase with time on task, reflecting the brain's fundamental need for periodic disengagement from external tasks (Lim & Dinges, 2008). In a similar vein, the cognitive load theory, developed by Sweller, Van Merrienboer, and Paas (1998), offers the perspective that our working memory has severe limitations in both capacity and duration. Since sustained attention tasks invariably impose some cognitive load, these fundamental working memory limitations make it impossible to maintain perfect performance indefinitely, regardless of motivation or effort.

From a somewhat related perspective, goal‐activation theory proposed by West, Murphy, Armilio, Craik, and Stuss (2002) suggests that maintaining task goals requires periodic *cognitive refreshing*, without which goal neglect naturally occurs. This necessity for periodic goal reactivation implies that truly continuous task focus is impossible—there must be moments when attention briefly shifts to refresh goal representations. While acknowledging the role of voluntary control, motivation theories of attention further note that even under conditions of maximum motivation, such as life‐threatening situations, humans cannot indefinitely maintain perfect attention.

The opportunity cost model proposed by Kurzban, Duckworth, Kable, and Myers (2013) suggests that our cognitive systems continuously evaluate the costs and benefits of maintaining attention on a given task, making some degree of attention shifting inevitable even when the stakes are high. The role of neuro‐modulatory systems adds another layer to this point. Research on the cholinergic system shows that sustained attention requires persistent activation of cholinergic neurons in the basal forebrain (M. Sarter et al., 2016). Studies of the noradrenergic system demonstrate that optimal attention requires patterns of firing in locus coeruleus neurons (Aston‐Jones & Cohen, 2005). These biological and cognitive constraints operate synergistically, creating multiple, overlapping limitations on sustained attention capability. The evidence suggests these limitations reflect fundamental aspects of neural organization rather than simply performance limitations that could be overcome through training or motivation.

## 5 Apparent counterexamples and their analysis

While the evidence suggests the theoretical impossibility of perfect sustained attention, several research areas and real‐world examples appear to challenge this conclusion. The study of “flow states” presents an apparent contradiction, with individuals reporting intense focus for extended periods without typical vigilance decrements (Csikszentmihalyi, 1997; Weber, Tamborini, Westcott‐Baker, & Kantor, 2016). For instance, studies of elite military personnel (e.g., Matthews, Warm, Shaw, & Finomore, 2019) and expert meditators (e.g., Lutz et al., 2009) have shown remarkable capabilities for sustained attention under extreme conditions. Matthews et al. (2019) demonstrated that, through intensive training, U.S. Army Rangers and other elite soldiers can maintain high vigilance performance for periods exceeding typical limits. Similarly, research on experienced Buddhist monks reveals they can sustain attention and reduce mind‐wandering for extended periods during meditation (Lutz et al., 2009; Tang & Posner, 2009). However, it should be acknowledged that the literature often reports that even elite performers exhibit measurable fluctuations and decrements when examined closely (Fiore, Jentsch, Bowers, & Salas, 2017; Mrazek, Franklin, Phillips, Baird, & Schooler, 2013).

Additionally, pharmaceutical cognitive enhancers like modafinil have been shown to reduce vigilance decrements and improve sustained attention in both sleep‐deprived and well‐rested individuals (Repantis, Schlattmann, Laisney, & Heuser, 2010). Neurofeedback training has also demonstrated the plasticity of attention networks, enabling individuals to voluntarily regulate their attention and improve performance (de Bettencourt, Cohen, Lee, Norman, & Turk‐Browne, 2015). However, closer examination suggests these counterexamples represent optimized attention management rather than truly overcoming the fundamental limitations of sustained attention. Cognitive enhancers and neurofeedback provide tools for managing attention, not eliminating its underlying constraints. The flow state experiences may, instead, involve periodic shifts in attention rather than perpetual vigilance. Ultimately, the theoretical impossibility of perfect sustained attention remains a fundamental constraint.

## 6 Practical implications for safety‐critical systems

If perfect sustained attention remains a theoretical impossibility, this then raises rather serious concerns about safety‐critical systems that rely primarily or exclusively on human vigilance. Recent research, for example, has shown that lifeguards experience a rapid decline in drowning detection performance as observation time increases, regardless of their experience level or cognitive abilities (Sharpe et al., 2024, 2023). Even highly trained lifeguards exhibit significant drops in vigilance within just 10 min of continuous monitoring, resulting in potentially critical lapses in surveillance. Furthermore, the aviation industry provides additional compelling evidence of the risks associated with relying on human sustained attention. A comprehensive analysis by the National Transportation Safety Board (NTSB, 2017) in the United States found that vigilance failures contributed to approximately 20% of aviation incidents, even in situations where multiple crew members were present. These findings suggest that some current practices and environmental controls are insufficient to mitigate the fundamental limitations of human attention.

In a similar vein, industrial safety research by N. B. Sarter and Woods (1995) has demonstrated that even in high‐stakes environments such as nuclear power plants and chemical processing facilities, operators invariably experience attention lapses that could have catastrophic consequences. Sarter and Woods argued that continuing to rely primarily on human vigilance in such settings represents a fundamental misconception of human cognitive capabilities and an unacceptable safety risk. The security industry faces similar challenges, with studies of CCTV operators showing significant decrements in threat detection performance over time (Donald & Donald, 2015). Even when operators are aware of the critical nature of their task and highly motivated to maintain attention, they seemingly cannot overcome the biological constraints that make perfect vigilance impossible. This has serious implications for how we approach security monitoring and surveillance.

## 7 Optimizing human–technology balance

The critical challenge for occupational research moving forward lies not in attempting to overcome the impossibility of perfect sustained attention, but rather in determining the optimal balance between human capabilities and technological support across different task domains. Wickens' multiple resource theory (2008) provides a useful framework for understanding how different types of tasks draw upon distinct attentional resources, suggesting that the appropriate human–technology balance may vary significantly depending on the specific demands of each task. Recent research in air traffic control has begun to map out this balance, identifying specific phases of operations where human operators outperform automated systems and others where technological support becomes crucial (Lundberg & Johansson, 2021; Svensson, 2020). In general, the findings of such studies (e.g., Lundberg & Johansson, 2021; Svensson, 2020) suggest that humans excel in tasks requiring contextual understanding, pattern recognition, and adaptive decision‐making during normal operations, while automated systems prove superior for maintaining vigilance during routine monitoring and detecting subtle deviations from expected parameters.

To provide some additional examples from industry, the maritime sector has provided some valuable insights on human attention performance over time through studies of bridge automation systems. Research by Hetherington, Flin, and Mearns (2020) demonstrated that human operators remain superior to automated technological systems in complex navigation scenarios requiring integration of multiple information sources and anticipation of other vessels' behaviors. However, Hetherington et al. also showed that sustained monitoring of instrument displays and environmental conditions is better handled by automated systems, with humans serving in a supervisory capacity to interpret and act upon significant deviations. In the medical field, for example, Andrade et al. (2020, 2021) have developed a framework for identifying the “sweet spot” in human–technology collaboration during patient monitoring tasks. Research indicates that while automated systems excel at continuous vital sign monitoring and early warning detection, human clinicians remain essential for interpreting the clinical significance of changes and understanding the broader patient context. This suggests a model where technology supports rather than replaces human attention, allowing healthcare workers to focus their limited attention resources on tasks that require human expertise.

Likewise, studies of industrial process control by Vicente and Burns (2021) have identified specific attention thresholds at which human operators should transition from direct control to technology‐supported monitoring. Their work suggests that operators can effectively maintain direct control for periods of up to 45 min before vigilance decrements become significant enough to warrant increased technological support. This type of precise threshold identification represents a promising direction for future occupational research. Indeed, this *operator limit* has also been noted in security surveillance research which has begun to quantify the optimal rotation periods for human operators and the specific conditions under which automated detection systems should take primary responsibility for monitoring (Bor & Koech, 2023; De Bruyne et al., 2023). Findings tentatively suggest that human operators should maintain primary monitoring responsibility during periods of high activity or unusual events, while automated systems should handle routine surveillance during low‐activity periods.

It is readily apparent that manufacturing environments have provided some valuable cognitive science insights on human attention processes and limitations through studies of quality control processes. Research repeatedly demonstrates that humans outperform automated inspection systems in detecting novel or unexpected defects, while automated systems excel at maintaining consistent detection of known defect types over extended periods (e.g., Banik & Dandyala, 2019). A critical appraisal of the line of research outlined above suggests that a hybrid approach would have the greatest efficacy, where automated systems handle routine inspection tasks while human operators focus their attentional resources on addressing anomalies and updating key detection criteria.

The development of *adaptive automation systems*, as described by Parasuraman (2020), represents a promising direction for achieving this optimal balance. These systems dynamically adjust the level of automation based on real‐time measurements of operator workload and attention state, effectively creating a fluid partnership between human and technological capabilities. This approach acknowledges both the strengths and limitations of human attention while ensuring that technological support is deployed when and where it is most needed. Future occupational research should focus on developing more precise metrics for determining these transition points between human and technological primacy. Montano (2011) suggested that such research should consider not only task characteristics and time‐on‐task effects but also environmental factors, operator expertise, and the potential consequences of errors. This comprehensive approach would help establish evidence‐based guidelines for the implementation of human–technology partnerships across different occupational settings.

## 8 Cross‐cultural and individual differences

Cross‐cultural research provides another compelling perspective on the universality of attention limitations while highlighting different approaches to managing them. Tang and Posner's (2009) seminal work on attention training across cultures reveals that while traditional practices like meditation may enhance attention regulation, they do not eliminate the basic constraints on sustained attention. Supporting this, Mrazek et al. (2013) demonstrated that even experienced meditation practitioners show inevitable fluctuations in attention, though they may become more adept at recognizing and recovering from lapses. These findings suggest an implicit understanding of attention's natural rhythms that modern workplace designs often ignore.

Individual differences in attention capabilities provide another important perspective on the impossibility of perfect sustained attention (e.g., Unsworth et al., 2021). Kane, Conway, Hambrick, and Engle (2007) conducted extensive research on working memory capacity and attention control, revealing substantial individual variations in the ability to maintain focused attention. However, even individuals at the highest end of the attention performance spectrum show inevitable vigilance decrements and attention lapses. Engle's (2018) review of working memory and attention research demonstrates that these individual differences affect the rate and magnitude of attention decline rather than eliminating the fundamental limitation itself. The denouement from such reviews of the literature on working memory and attention performance is clear that while selection and training can optimize attention performance within certain bounds, they cannot overcome the basic constraints of human cognitive architecture.

## 9 Economic implications and future directions

The economic implications of attention limitations provide a compelling argument for investing in appropriate technological support systems. Swanson, Holton, and Holton (2011) analyzed the costs of attention‐related errors in healthcare settings, finding that vigilance failures contributed significantly to medical errors, with associated costs exceeding $20 billion annually in the U.S. healthcare system alone. Complementing this, Reason's (2000) framework for managing organizational accidents emphasizes how systemic approaches to error prevention, including technological support systems, prove more cost‐effective than attempting to eliminate human error through training alone. These economic realities suggest that continuing to rely primarily on human sustained attention is not only theoretically flawed but also financially unsound.

However, emerging technologies offer new possibilities for managing attention limitations without eliminating them entirely. Zander and Kothe's (2011) comprehensive review of brain–computer interfaces for workload detection shows how future systems might provide more sophisticated support for human attention limitations. Matthews, Reinerman‐Jones, Barber, and Abich (2015) further demonstrated how adaptive automation systems can effectively support attention management in complex operational environments. Rather than attempting to eliminate attention constraints, these technologies work by better detecting and predicting attention states, allowing for more dynamic and proactive support. These findings align with our theoretical understanding of attention's fundamental limitations while offering practical paths forward for safety‐critical operations.

Even these advanced technologies operate within the framework of managing rather than eliminating attention limitations. The development of these systems reflects a growing recognition that the goal should not be to achieve perfect sustained attention but rather to create more sophisticated ways of working within our cognitive constraints. This aligns with Hancock and Warm's (1989) adaptive‐resource theory, which remains influential in understanding how humans manage attention resources in complex task environments. The integration of developmental insights, cultural perspectives, economic realities, individual differences, and technological possibilities points toward a future where we design systems that work in harmony with human attention limitations rather than fighting against them. This comprehensive view suggests that our historical approach of trying to maintain sustained attention through willpower and training alone has been fundamentally misguided. Instead, we need integrated approaches that acknowledge both the impossibility of perfect sustained attention and the various ways we can work within and around these limitations.

## 10 System and environment considerations

The theoretical impossibility of perfect sustained attention necessitates thoughtful system design that effectively detects, prevents, and accommodates attentional limitations. Several promising approaches might allow systems to detect attentional lapses before they result in performance decrements. Eye‐tracking technology may identify reduced scanning, prolonged fixations, or increased blink rates associated with vigilance decrements (Di Stasi et al., 2016; Sharpe & Smith, 2024). Pupillometry might provide insights by tracking pupil diameter changes linked to cognitive load and attention (van der Wel & van Steenbergen, 2018). Neurophysiological methods, such as portable EEG detecting alpha and theta wave shifts (Huang et al., 2018) and Functional Near‐Infrared Spectroscopy (fNIRS) monitoring prefrontal activity (McKendrick, Parasuraman, & Ayaz, 2015), could offer direct indicators of attentional state. Behavioral markers, including response time variability, error patterns, and micromovements, may also signal declining attention (Körber, Cingel, Zimmermann, & Bengler, 2015). Machine learning might be able to integrate these diverse data streams, potentially improving predictive accuracy by accounting for individual attentional patterns (Acı, Kaya, & Mishchenko, 2019). However, it is important to test these methods explicitly before implementation to ensure their reliability and practical effectiveness. Without rigorous validation, these systems may not perform as intended in real‐world settings. As technology advances, less invasive neural monitoring may become feasible, allowing for the development of more practical, integrated detection systems.

When addressing how systems should respond to detected attentional lapses, two general approaches may emerge. Warning‐based systems alert the human operator to potential attention decrements, while autonomy‐based systems temporarily assume control of certain functions. In situations with moderate risk, a graduated warning system might be appropriate. Such a system could begin with subtle cues that become more explicit if attention continues to decline (e.g., Wiese & Lee, 2004). This approach maintains human agency while providing necessary support. For high‐risk scenarios, systems might need to assume certain functions without requiring human acknowledgment. This approach parallels higher levels of vehicle autonomy, where systems take over critical functions when human attention proves inadequate. The key consideration is balancing immediate safety with long‐term skill maintenance. The timing of any intervention likely affects its efficacy. Systems that can predict attention decrements might prove more effective than those responding only after performance has already declined. However, such predictive systems would need to carefully balance sensitivity against the risk of unnecessary interventions. A potential concern with any automated assistance is the development of over‐reliance (Parasuraman & Manzey, 2010). Systems designed to compensate for attentional limitations should ideally avoid creating dependency that further erodes attention capabilities. Dynamic adjustment of assistance levels might help maintain an appropriate level of human engagement (e.g., Chen, Lv, Qiang, & Liu, 2024).

Beyond detection and prevention, systems could be designed to fundamentally accommodate attentional limitations. Task restructuring represents one possible approach. Rather than requiring sustained vigilance, work might be organized into shorter attention intervals interspersed with different activities (see Langner & Eickhoff, 2013; Wahn & König, 2017 for review). Various technologies might reduce attentional demands by transforming information into more readily processed forms. Visual augmentation that highlights critical information, conversion of data into sound patterns, or tactile feedback systems could potentially maintain awareness while reducing cognitive load. Another approach might involve providing support precisely when attention is likely to flag. Rather than attempting to maintain continuous attention, systems could offer enhanced information and decision support during predicted vulnerability periods. This approach would work with natural attention rhythms rather than against them. Collaborative systems might also prove valuable. Distributing vigilance responsibilities across multiple humans, or between humans and automated systems, could compensate for individual attentional limitations. The challenge would lie in maintaining effective coordination and shared awareness.

The recognition of attention's fundamental limitations suggests a reconsideration of task allocation between humans and automated systems. A thoughtful approach might assign continuous vigilance tasks to automated systems (e.g., drowning detection systems) while reserving human attention for activities requiring creativity, contextual understanding, and moral judgment (e.g., distinguishing between intentional breath holds and drowning). This reallocation would require careful consideration of the human–system boundary. Complete removal of humans from monitoring loops could create vulnerability to automation failures, while excessive demands for vigilance would inevitably lead to attentional lapses. The appropriate balance would likely vary by context. Physical environment design might further support attentional management. Factors such as lighting patterns, acoustic properties, and spatial organization could potentially influence sustained attention capacity (e.g., Green, Cohen‐Zion, Haim, & Dagan, 2017). Environmental design could possibly facilitate natural attentional refreshment through appropriate sensory stimulation and opportunities for microbreaks. Future environments might even incorporate spaces specifically designed to support different attentional states throughout the workday. Such environments would recognize that different types of attention (focused, sustained, divided, selective) might benefit from different environmental conditions. The ultimate goal should not be to eliminate attentional limitations—apparently an impossible task—but rather to create conditions where these limitations pose minimal risk while maximizing the unique capabilities of human cognition. This approach acknowledges the theoretical impossibility of perfect sustained attention while seeking practical systems that function effectively within the constraints of human cognitive architecture.

## 11 Conclusion

The theoretical impossibility of perfect sustained attention emerges from multiple converging lines of evidence: evolutionary considerations, neural mechanisms, cognitive architecture, and fundamental biological constraints. This impossibility is not a failure of human capability but rather reflects the adaptive design of our cognitive systems. Understanding and accepting this theoretical impossibility should inform the design of human systems, from education to workplace safety, leading to approaches that work with, rather than against, the fundamental properties of human attention.
